# Age-related reference intervals for thyroid function tests are beneficial in adults over 60 years

**DOI:** 10.1177/00045632261426852

**Published:** 2026-02-09

**Authors:** George F. G. Allen, Sarah Blampied, Kashyap A. Patel, Timothy J. McDonald, Andrew T. Hattersley, Bijay Vaidya

**Affiliations:** 1 NIHR Exeter Biomedical Research Centre, University of Exeter Medical School, Exeter, UK; 2 Blood Sciences, 9553 Royal Devon University Healthcare NHS Foundation Trust, Exeter, UK; 3 Department of Clinical and Biomedical Sciences, Faculty of Health and Life Sciences, 151673 University of Exeter, Exeter, UK; 4 NIHR Exeter Clinical Research Facility, 9553 Royal Devon University Healthcare NHS Foundation Trust, Exeter, UK; 5 Department of Endocrinology, Royal Devon & Exeter Hospital, University of Exeter Medical School, Exeter, UK

**Keywords:** Thyroid reference intervals, TSH, FT4, age-related, elderly, older adult

## Abstract

**Background:**

The rise in serum thyroid stimulating hormone (TSH) in healthy older adults is well established. However, age-related reference intervals are not widely used. We aimed to establish all-adult and age-related reference intervals for TSH, free thyroxine (FT4) and free triiodothyronine (FT3) and determine their impact on primary care thyroid testing results.

**Methods:**

We measured TSH, FT4 and FT3 by Roche Cobas assays in a reference cohort of 1364 anti-thyroid peroxidase antibody negative adults with no self-reported medical conditions or medications. Reference intervals were generated for all-adult, 18–60 and over 60 years. Reference intervals were applied to 21,286 primary care tests with no known thyroid disease to assess effect on test interpretation.

**Results:**

In the reference cohort, 23.2% were over 60 years compared to 50.4% of those undergoing thyroid testing in primary care. With an all-adult reference interval, 8.2% of over 60s had an elevated TSH, 7.0% were classified as subclinical hypothyroid and 0.8% as overt hypothyroid. With age-related reference intervals, this fell to 4.4% with an elevated TSH, 3.7% subclinical hypothyroid and 0.4% overt hypothyroid. Minimal changes were seen in the 18–60 years group.

**Conclusions:**

An all-adult reference interval derived from healthy and therefore younger individuals is less appropriate for the older subset of the population being tested. Application of an age-related reference interval for over 60s would reduce the proportion of patients with abnormal thyroid test results. In turn, this would decrease potentially unnecessary cascade and repeat testing as well as regular follow-up in primary care.

## Introduction

The appropriateness of reference intervals (RI) for serum thyroid stimulating hormone (TSH) and free thyroxine (FT4) has been much debated due to their role in defining subclinical and overt disease and as thresholds for cascade and follow-up testing.^1–3^ The upper reference limit of TSH is particularly controversial as a threshold for defining subclinical hypothyroidism, with arguments to either lower or increase the reference limit being made.^1^ Others have recommended adopting a separate decision limit based on risk of progression.^3^ The TSH RI also impacts laboratory workload as results outside the RI are used as a threshold for cascade FT4 testing, confirmatory repeat testing of abnormal results and subsequent regular follow-up testing.^4,5^

Reference intervals are commonly derived from samples of healthy individuals who are likely to be younger and so less representative of the subset of the population routinely being tested. Healthcare services are more frequently accessed by people of older age than younger adults.^6^ Likewise, blood tests are more commonly requested in older adults.^7,8^ Conversely, reference intervals have classically been derived from cohorts of healthy people with some reference interval studies actively excluding older adults from the reference sample.^9,10^ Even without exclusion, the increasing burden of chronic disease with age will naturally lead to the recruitment or selection of younger individuals to produce a healthy reference sample.^11^

There are age-related changes in TSH and FT4 that indicate a need for specific RIs for older adults, but these have not been widely adopted despite high rates of testing in this age group. An age-related rise in TSH is well-established whilst some studies also indicate a fall in FT4.^8,12–14^ This is an adaptation to older age which is not associated with thyroid autoimmunity^15^ and does not benefit from treatment.^16,17^ Age-related RIs have been proposed for older adults, but to our knowledge these have not been widely adopted.^5,18–22^

Given the high rate of thyroid testing in older people and the physiological changes in TSH and FT4, we hypothesised that introduction of age-related RIs would improve thyroid test result classification for patients routinely being tested. Our aim was to derive local adult age-related thyroid RIs on Roche Cobas assays and determine the effect of using these on abnormal thyroid test result classification in primary care.

## Methods

### Reference cohort selection

A reference cohort of healthy volunteers was selected from participants recruited to the Exeter 10,000 study with samples stored in the Peninsula Research Bank.^23^ Recruitment of volunteer participants over 18 years of age to the Exeter 10,000 study has been described previously.^24^ The Exeter 10,000 study has been approved by the South West Research Ethics Committee of the NHS Health Research Authority (19/SW/1059). Use of samples and participant data for the current study was approved by the Peninsula Research Bank Steering Committee.

Participants were selected from the Peninsula Research Bank based on answers to a health questionnaire completed at recruitment that included a list of all medical conditions and current medications. 750 female and 750 male participants were consecutively selected with no self-reported diagnosis of any medical condition or any current treatment. Serum samples were stored frozen at −70°C or below in the Peninsula Research Bank from time of recruitment.

### Clinical cohort selection

A clinical cohort was used to assess the impact of the RIs generated from the reference cohort. The clinical cohort used routinely collected clinical test results data from samples analysed at the Royal Devon University Healthcare NHS Foundation Trust. TSH and FT4 records from primary care requests were extracted for a 6-month period between 1st January 2023 and 30th June 2023.

Clinical laboratory test data was extracted from the electronic patient record (EPIC systems, Wisconsin, USA). Data was extracted by clinical laboratory professionals as part of routine service development, and all data were anonymised prior to analysis. Patients under 18 years were excluded. Patients with known thyroid disease were excluded based on clinical details provided at request. The GP ordering system includes both a box to provide clinical details and a compulsory drop down list of reasons for TSH request including hypothyroidism, hyperthyroidism, hypopituitarism, thyroid cancer, prescribed amiodarone, no diagnosed thyroid problem or an additional ‘other’ option that requires further information. Patients were therefore excluded if any of the following terms were present in the clinical details provided: levothyroxine, hypothyroid, Hashimoto, Graves, hyperthyroid, carbimazole, lithium, PTU, hypopituitarism, thyroid cancer or amiodarone. A compulsory yes/no question on pregnancy for females was used to identify and exclude pregnant patients. Finally, to exclude those with thyroid disease and missing or inaccurate clinical details, all patients with a request for TSH within the prior 2 years were excluded. To ensure each test result was from a unique patient, repeat measurements within the 6-month period were removed.

### Sample analysis

Reference cohort samples were thawed for 2 h at room temperature and mixed prior to analysis. Clinical cohort samples were received from primary care via ambient temperature transport and typically analysed within 4 hours of receipt within the laboratory. All samples in the reference cohort were analysed for TSH, FT4 and FT3 and all clinical cohort samples were analysed for TSH. For the clinical cohort, manufacturer-defined RIs were applied during routine clinical practice, and FT4 was analysed on all samples with a TSH value outside of the RI. Manufacturer RIs were TSH 0.27 to 4.2 mIU/L, FT4 12.0 to 22.0 pmol/L and FT3 3.9 to 6.8 pmol/L. For the reference cohort, anti-thyroid peroxidase (anti-TPO) antibodies were measured in all subjects to allow exclusion of subjects positive for these antibodies. Anti-TPO antibodies were defined as positive based on the manufacturer defined cut-off of greater than 34 IU/mL.

Analysis was performed on a Roche Cobas e801 analyser with the following electrochemiluminescence immunoassays: Elecsys TSH, Elecsys FT4 III, Elecsys FT3 III and Elecsys Anti-TPO (Roche Diagnostics, Burgess Hill, UK). At the time of testing, within-laboratory % CV for TSH was 2.7%, FT4 3.3%, FT3 2.6% and anti-TPO 6.7%.

### Thyroid function tests result classification

Results were classified as overt hypothyroid where TSH was above the upper limit of the TSH RI and FT4 was below the lower limit of the FT4 RI. Results were classified as subclinical hypothyroid where TSH was above the upper limit of the TSH RI and FT4 was within the FT4 RI. Overt hyperthyroidism was classified as TSH below the lower reference limit of the TSH RI and FT4 or FT3 above respective upper reference limits. Subclinical hyperthyroidism was classified as TSH below the lower reference limit of TSH RI with FT4 and FT3 within respective reference intervals.

### Statistical analysis

Statistical analysis was performed using R.^25^ Normal distribution was assessed in the reference cohort using the Shapiro–Wilk test and in the clinical cohort the Lilliefors test due to the large sample size. Where *P* < .05 on normality test, non-parametric tests and summary statistics were used. D’Agostino’s test was used to assess skewness. Individual comparisons between medians were made using the Wilcoxon rank-sum test. Multiple comparisons were made using the Kruskal–Wallis test followed by Dunn’s post hoc test with Bonferroni correction. Result categorisation of paired data was assessed with the McNemar test with continuity correction. RIs were generated from the 2.5th and 97.5th centiles of untransformed data in accordance with Clinical Laboratory Standards Institute guidance,^26^ and anti-TPO antibody positive individuals were excluded as per US National Academy of Clinical Biochemistry guidance.^27^ No outliers were removed during RI generation. Non-parametric bootstrap confidence intervals were generated for 2.5th and 97.5th centiles from resampling with 1000 repetitions using the R boot package.^28,29^ Figures were produced in R using ggplot2.^30^

## Results

### Characteristics of the reference and clinical cohorts

Bio-banked samples from 750 females and 750 males with no diagnosed disease or medication were analysed for anti-TPO antibodies. Of this cohort, 9% (136/1500) were anti-TPO antibody positive, of which 67% were female. After exclusion of anti-TPO antibody positive individuals, the final reference cohort included 1364 individuals and was 48% female.

**Figure 1.**
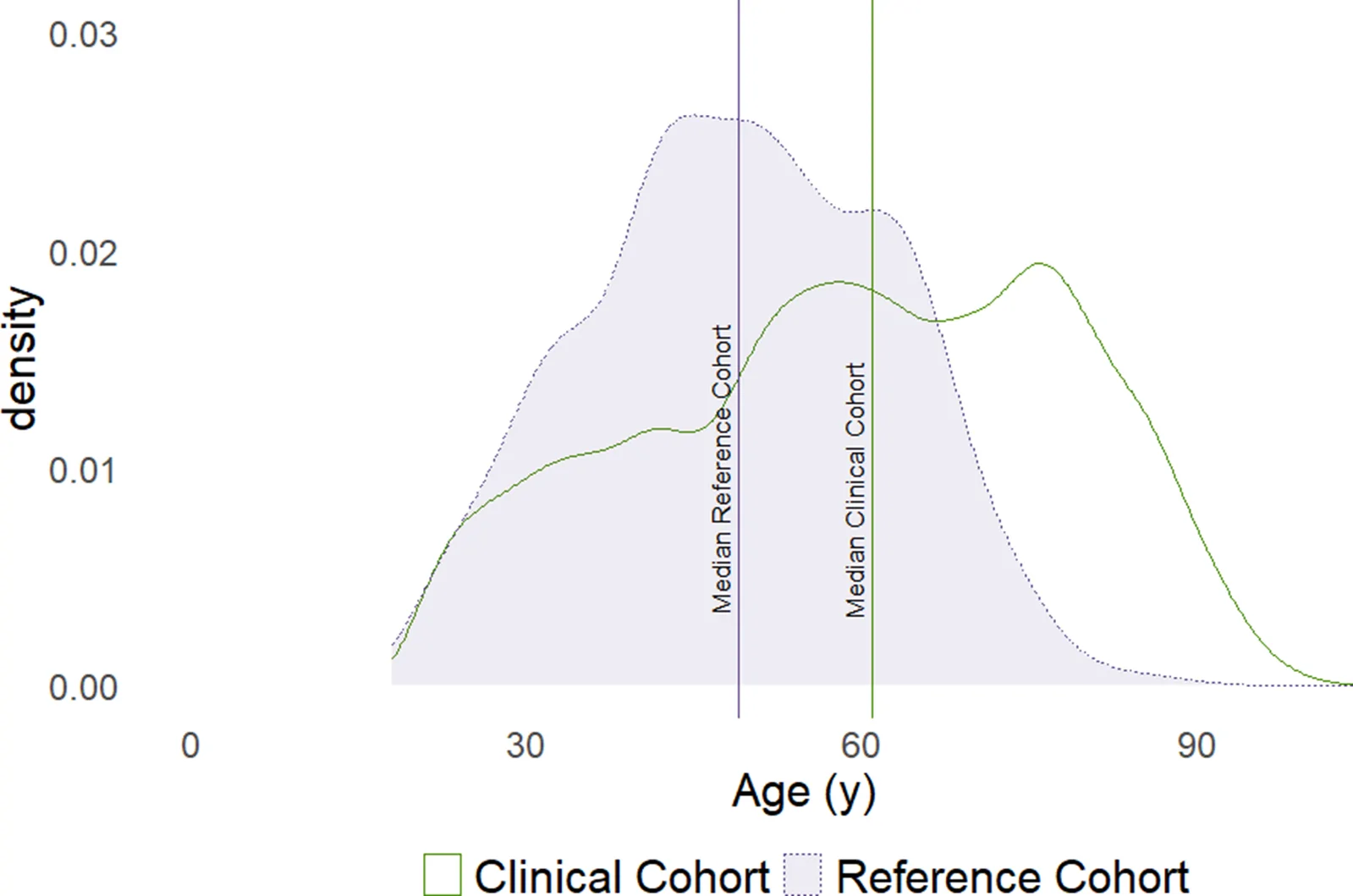
Age density plot of the reference cohort (n = 1364) of healthy adult volunteers with no medical conditions or medications and the clinical cohort (n = 21,286) of adults in primary care having thyroid tests. Patients with thyroid disease, thyroid-related medication, medication known to affect thyroid or who were pregnant were excluded from the clinical cohort.

After exclusions, the clinical cohort of routine primary care thyroid test results included 21,286 patients with either no thyroid disease or previously undetected thyroid disease, of which 64.5% were female. The clinical cohort had a median age of 61 years (IQR 46–75) which was older than the reference cohort with a median age of 49 years (IQR 40–60, *P* < .0001). The age distribution of the clinical cohort was negatively skewed (skew −0.216, *P* < .0001) with 50.4% of patients over the age of 60 years (see Figure 1). The age distribution of the reference cohort was minimally skewed (skew −0.005, *P* = .9321) with 23.2% of patients over the age of 60 years.

### Effect of age on thyroid test results

The clinical cohort was partitioned into age groups 18–30, 31–40, 41–50, 51–60, 61–70, 71–80 and over 80. A significant increase in median serum TSH concentration was seen in all age groups above 60 years in comparison to younger age groups of 60 years and below (see supplemental Figure 1 and supplemental table 1).

**Figure 2.**
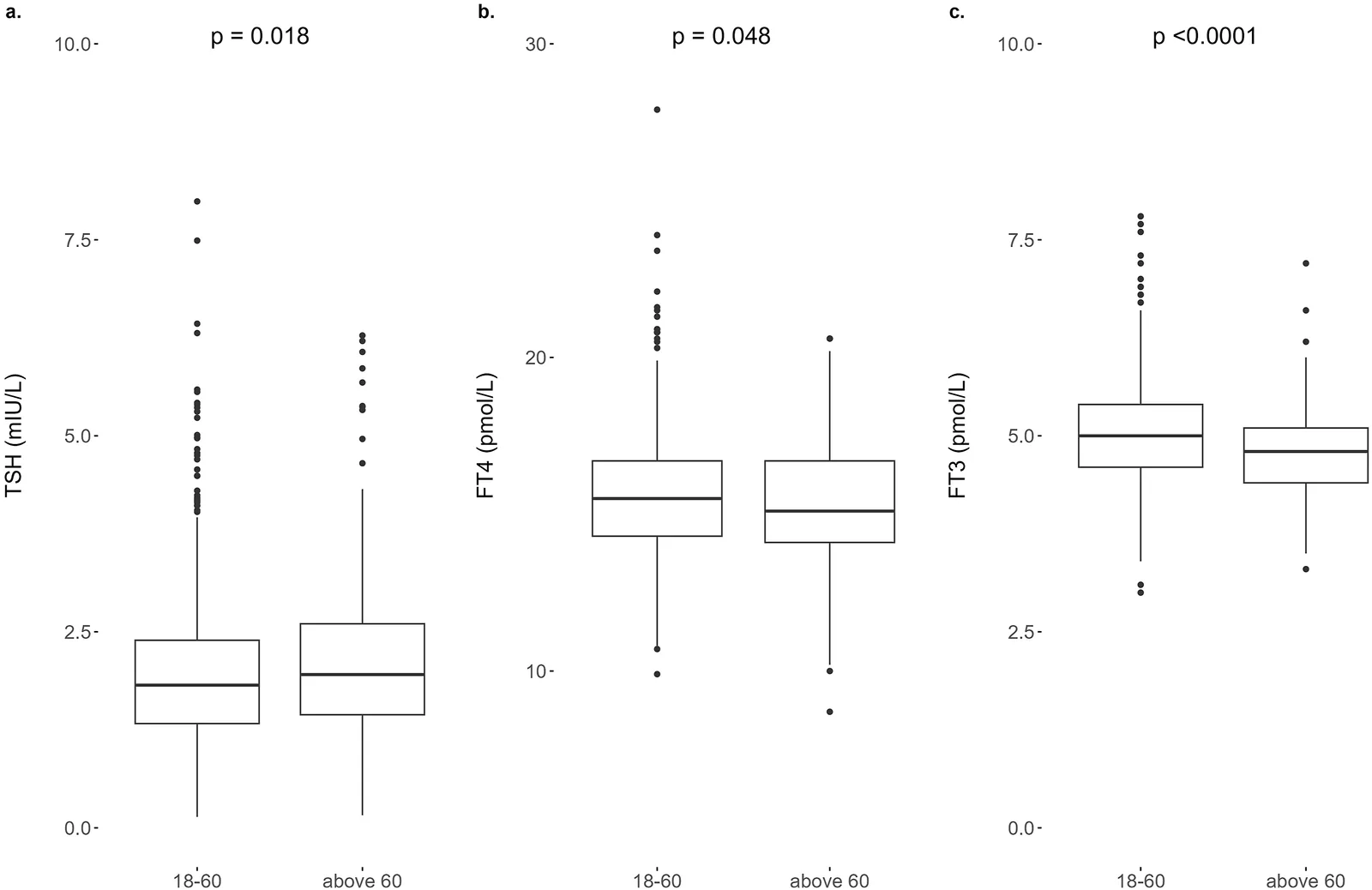
Effect of age on thyroid function test results in the reference sample: (a) TSH (mIU/L), (b) FT4 (pmol/L) and (c) FT3 (pmol/L). Group 18–60 years n = 1048; group above 60 years n = 316. Statistical comparison by Wilcoxon sum rank test.

Given the difference in TSH observed in the clinical cohort with age, we decided to divide the reference cohort into two age groups 18–60 years and over 60 years reflecting the median of the clinical cohort of 61 years. In the 18–60 years group, median TSH was lower at 1.82 mIU/L (IQR 1.33–2.39) than above 60 years at 1.96 mIU/L (IQR 1.44–2.60, *P* = .0182, see Figure 2). Median FT4 was higher at 15.5 pmol/L (IQR 14.3–16.7) at age 18 to 60 years than above 60 years at 15.1 (IQR 14.1–16.7, *P* = .0481). Median FT3 was higher at 5.0 pmol/L (IQR 4.6–5.4) at age 18–60 years than above 60 years at 4.8 (IQR 4.4–5.1, *P* < .0001). These results confirm an impact of age on thyroid function supporting that age-related RIs are appropriate.

### Effect of sex on thyroid test results

In the clinical cohort, median TSH was 1.77 mIU/L (IQR 1.22–2.57) in females and 1.85 mIU/L (IQR 1.30–2.63, *P* < .0001) in males. The apparent effect of sex on serum TSH was not observed in the reference cohort where median TSH was 1.85 mIU/L (IQR 1.28–2.48) in females and 1.88 mIU/L (IQR 1.41–2.42, *P* = .3174) in males. When the reference cohort was further partitioned into the age groups 18–60 and over 60, median TSH was not significantly different between males and females at either age group (see supplemental table 2). The lack of a consistent effect of sex on median TSH did not support the use of sex-specific reference intervals.

The effect of sex on FT4 and FT3 in the clinical cohort could not be reliably determined as these tests were only analysed when TSH was outside of current reference interval (FT4) or suppressed below 0.1 mIU/L (FT3). In the reference cohort, median FT4 was lower in females at 15.2 pmol/L (IQR 14.1–16.4) than males at 15.6 pmol/L (IQR 14.4–17.0, *P* < .0001). Likewise, median FT3 was lower in females at 4.6 pmol/L (IQR 4.6–5.0) than males at 5.2 pmol/L (IQR 4.8–5.6, *P* < .0001). Age-stratified reference intervals for FT4 and FT3 were similar between the sexes and are presented in supplemental table 3.

### Age-related adult reference intervals

We generated age-related RIs for TSH, FT4 and FT3 from the reference cohort for all adults, and then separately for 18–60 years and over 60 years using 2.5th and 97.5th centiles (see Table 1). The clinical cohort was partitioned into two corresponding groups of 18–60 years and over 60 years to allow application of the RIs.

**Table 1.** Reference Intervals for Serum Thyroid Stimulating Hormone (TSH), Free Thyroxine (FT4) and Free Triiodothyronine (FT3) for all Adults (Over 18 Years) and by Age Group With Bootstrap 95% Confidence Intervals.

Age group	n	TSH (mIU/L)		FT4 (pmol/L)		FT3 (pmol/L)	
		25th Centile (95% CI)	97.5th Centile (95% CI)	2.5th Centile (95% CI)	97.5th Centile (95% CI)	2.5th Centile (95% CI)	97.5th Centile (95% CI)
All adult	1364	0.65 (0.58, 0.70	4.30 (4.11, 4.83)	12.0 (11.5, 12.3)	19.4 (19.0, 19.8)	3.9 (3.8, 4.0)	6.3 (6.2, 6.5)
18–60	1048	0.66 (0.60, 0.72)	4.17 (3.85, 4.57)	12.3 (11.9, 12.4)	19.5 (19.0, 19.8)	3.9 (3.8, 4.0)	6.4 (6.2, 6.5)
Above 60	316	0.60 (0.44, 0.74)	5.34 (4.19, 0.67)	11.2 (10.8, 12.0)	18.7 (18.4, 19.6)	3.8 (3.8, 4.0)	5.9 (5.7, 6.0)

### Age-related RIs in the over 60s greatly reduces those with an elevated TSH

**Figure 3.**
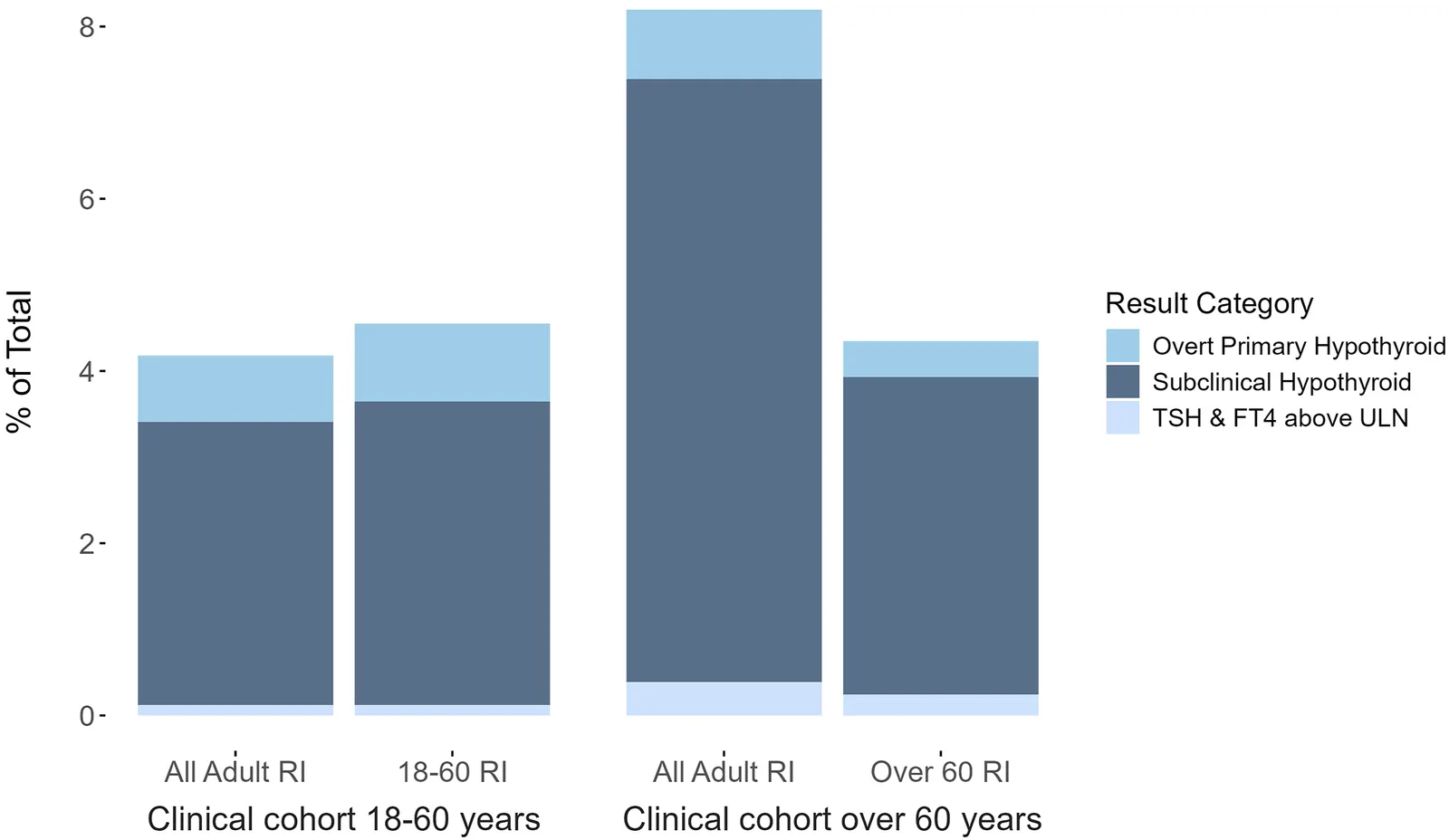
Categorisation of thyroid function tests following application of all-adult and age-specific reference intervals to clinical cohort 18–60 years (n = 10,531*) and over 60 years (n = 10,747). Results were classified as subclinical hypothyroid where TSH was above upper reference limit and FT4 was within reference interval and overt primary hypothyroid where TSH was above upper reference limit and FT4 was below lower reference limit. *Eight results were excluded from the 18–60-year cohort where TSH was above RI but FT4 was unavailable. RI: reference interval; ULN: upper limit of normal.

We went on to assess the impact of applying age-related RIs on routine data. The all-adult and age-related RIs from the reference cohort for TSH and FT4 were applied to the corresponding age groups from the clinical cohort. In the over 60 years group, 8.2% (882/10,747) had a TSH above the all-adult RI. Using the over 60s RI for TSH, this fell to 4.4% (468/10,747; *P* < .0001; see Figure 3). In the 18–60 years group, 4.2% (440/10,539) had a TSH above the all-adult RI which rose to 4.6% (479/10,539; *P* < .0001) using the 18–60 RI.

We further partitioned the reference cohort to generate age-specific RIs for 18–30, 31–40, 41–50 and 51–60 years (see supplemental table 4). These were then applied to corresponding groups of the clinical cohort. Across these four groups, there was minimal effect on TSH above RI at 4.2% (446/10,539) in comparison to the all-adult RI at 4.2% (440/10,539, *P* = .0412, see supplemental table 5). Due to the limited number of participants in the reference cohort at age 71–80 years (n = 56) or age over 80 years (n = 8), it was not possible to further partition RIs for those over 60 years.

### Most of those reclassified as euthyroid by age-related RI were originally defined as subclinical hypothyroid using the all-adult RI

In the over 60 years group, a subclinical hypothyroid pattern was present in 7.0% (752/10,747) with the all-adult RIs but when the over 60s RIs were used, this dropped to 3.7% (396/10,747; *P* < .0001). Eight patients had a TSH above RI and no available FT4 in the 18–60 years group and so were excluded (final n = 10,531). In the 18 to 60 years group, 3.3% (346/10,531) had a subclinical hypothyroid pattern with the all-adult RIs. This increased to 3.5% (371/10,531; *P* < .0001) with the 18–60 RIs.

### Overt primary hypothyroid thyroid tests also decreased with use of age-related RIs in the over 60s

An overt primary hypothyroid pattern was seen in 0.8% (87/10,747) of the over 60 years group with the all-adult RIs and 0.4% (45/10,747; *P* < .0001) with the over 60s RIs. Of those that were reclassified, 88% had a normal FT4 of 11.2 pmol/L or above (55% were reclassified as subclinical hypothyroid and 33% as euthyroid) and 12% with an isolated low FT4. In the 18 to 60 years group, 0.8% (81/10,531) had an overt primary hypothyroid pattern with the all-adult RIs and 0.9% (95/10,531; *P* = .0005) had an overt primary hypothyroid pattern with the 18–60 RIs.

### The effect of age-related RIs on low TSH was more limited

In the over 60 years group, a TSH below the lower reference limit was observed in 5.7% (614/10,747) with the all-adult RI and 5.0% (538/10,747; *P* < .0001) with the over 60s RI. In the 18–60 years group, 4.2% (441/10,531) had a TSH below the lower reference limit with the all-adult RI and 4.4% (458/10,531; *P* = .0001) with the 18–60 RI. In the clinical cohort, FT4 was only analysed when TSH was below the current lower reference limit of 0.27 mIU/L. This means that TSH results between the new lower limits and 0.27 mIU/L did not have a corresponding FT4 result available, which prevented categorisation of results as subclinical or overt hyperthyroid.

The all-adult and age-related lower limits of TSH RIs were higher than the manufacturer’s RI lower limit and so would all increase the proportion of patient’s categorised with low TSH. Using the manufacturer’s lower reference limit for TSH of 0.27 mIU/L, 1.8% (195/10,747) of over 60s and 0.9% (93/10,531) of 18–60 year olds had a TSH below the lower reference limit.

## Discussion

Our study demonstrates that thyroid tests are most commonly performed in people over 60 years of age and that age-related RIs for this group may be beneficial to reduce potential over-classification of abnormal thyroid test results. We found that use of age-related RIs for TSH and FT4 for over 60-year-olds resulted in a 47% reduction in TSH results above RI and fewer patients categorised as subclinical or overt hypothyroid. Age-related RIs had a more limited effect on thyroid test result classification with an 11% increase in TSH above RI in those aged 18 to 60 years. A recent report from the Whickham Longitudinal Population Cohort Study found that for participants in their 70s without thyroid disease, the prevalence of subclinical hypothyroidism rose from 3.5 to 9.0% over 8 years.^14^ However, if age-associated RIs were used, prevalence remained at 2.0% throughout. These findings are particularly relevant due to the high number of thyroid tests performed in older people. In our primary care cohort, more than half of those having thyroid tests were over the age of 60 years. A recent review of thyroid test requests in South Tyne found that nearly 50% of 61 to 80-year-olds had thyroid tests requested in a year.^8^

Introduction of age-related RIs for those over 60 years would have a positive impact for laboratories and primary care by reducing potentially unnecessary testing and follow-up. In our study, application of age-related RIs for over-60-year-olds led to a 47% reduction in those classified as subclinical hypothyroid and a 48% reduction in those with a primary overt hypothyroid pattern, of which 88% had a marginal FT4 of 11.2 to 11.9 pmol/L. This reduction would have a beneficial effect as follow-up of abnormal thyroid tests can include repeat confirmatory testing, anti-thyroid peroxidase antibody testing, regular follow-up thyroid testing and clinical review in primary care.^4,5^ Previous research suggests that those with subclinical hypothyroidism that were re-classified as euthyroid were unlikely to benefit from treatment or monitoring.^16,17,31^ In randomised controlled trials, levothyroxine treatment did not improve hypothyroid-related symptoms in patients over 65 years with subclinical hypothyroidism and a TSH up to 20 mIU/L.^16,17^ In participants over 65 years of age with a subclinical hypothyroid pattern and a TSH of 4.5 mIU/L to 6.9 mIU/L, 1% progressed to overt primary hypothyroidism whilst 46% reverted to euthyroid status over 2 years.^31^ In comparison, in those with a TSH of 10 mIU/L or above, 10% progressed to overt hypothyroidism and 7% reverted to euthyroid status.

A relationship between thyroid tests and age is well established. Within our reference cohort, we found that TSH was highest and FT4 lowest in those over the age of 60 years. The Bussleton study demonstrated an excess of elevated TSH levels in participants over the age of 50 years with a standard upper TSH RI of 4.0 mIU/L.^13^ This study also demonstrated a decrease in FT4 levels with age. The US National Health and Nutrition Examination Survey (NHANES) III found a continuous rise in TSH with age in those without thyroid disease or risk factors for thyroid disease.^32^ The Whickham study followed participants with a mean age of 77 years and found a rise in TSH of 0.29 mIU/L over a median of 7.8 years.^14^ The association of higher TSH with older age has also been observed in data collected in routine practice.^8^

Despite the clear age-related changes in thyroid function tests, the use of age-related RIs is surprisingly more controversial. Our study demonstrates the potential benefits of age-related RIs for those over 60 years, including a 47% reduction in those classified as subclinical hypothyroid and decreased laboratory workload from cascade FT4 testing and repeat follow-up testing. The use of age-specific RIs for TSH and FT4 has been advocated, particularly for those over 60 years on the basis of over-classification with standard RIs.^18,20,21^ Conversely, it has also been argued that age-specific TSH RIs have minimal impact on re-classification except in those of 85 years and above.^33^

There is a potential for age selection bias when choosing healthy participants that may make derived RIs less applicable to those being tested clinically. Our reference cohort only included those without any self-reported medical conditions or prescribed medications, which led to a cohort with a median age 12 years younger than those receiving thyroid tests in primary care. In the NHANES cohort, the proportion of healthy individuals decreased continuously with age and less than 20% of those over 60 years were classified as healthy.^11^ The frequency of primary care consultations in adults increases with age as does the frequency of common blood tests such as renal function or thyroid function tests.^6–8^ This systematic difference between reference interval samples and those receiving tests in primary care has the potential to result in reference intervals that are unsuitable for those routinely being tested.

A clinical decision limit based on risk may be more appropriate than a lower reference limit (LRL) for defining subclinical hyperthyroidism or for treatment monitoring. Our all-adult LRL is higher than the manufacturer’s LRL but consistent with previous studies.^10^ In comparison to the all-adult LRL, the over 60s LRL led to a 12% reduction in low TSH results. However, application of the manufacturer’s LRL resulted in 68% or 56% fewer low TSH results compared to the all-adult LRL or over 60s LRL, respectively. Moving from the manufacturer’s LRL would therefore increase mildly low TSH results as well as consequent cascade FT4 and follow-up testing. Categorising and monitoring additional patients with mildly suppressed TSH is unlikely to be beneficial. For subclinical hyperthyroidism, increased risk of adverse outcomes such as atrial fibrillation or progression to overt hyperthyroidism is only associated with TSH suppressed below 0.1 mIU/L.^34–36^ Similarly, in a primary care medical record analysis of patients on thyroid hormone replacement, an increased risk of mortality was observed when TSH was below 0.1 mIU/L but not at 0.1 to 1.0 mIU/L.^37^ This indicates that a clinical decision limit, such as 0.1 mIU/L, may be more appropriate to determine follow-up and additional testing.

The strengths of our study include the use of a large reference sample to allow partitioning and a dataset of routinely collected primary care thyroid tests to explore the consequences of derived RIs. The study used bio-banked samples that were prospectively collected from volunteers within the local population. Participants were selected who had no self-reported diagnosis of any disease or on any medication and those with anti-TPO antibodies were excluded. This provided a large number of reference individuals to generate RIs for TSH, FT4 and FT3 that were sufficient to allow age partitioning. We also made use of a large dataset of routinely collected primary care thyroid function tests from the same population as the reference sample to explore the consequences of the derived RIs for routine practice. Patients with known thyroid disease could be effectively removed from this dataset due to the robust questions asked at electronic ordering.

The weakness of our study is that derived RIs may not be broadly transferrable due to use of a single type of assay, the reference sample was from a single location and there were a small number of reference participants above 70 years. The RI and clinical results were produced with only one type of assay from Roche diagnostics. Given the known lack of standardisation between methods, especially for FT4, the derived RIs are unlikely to be applicable to other assays.^10^ The reference sample used participants recruited from the local population within the South West of England. This could potentially limit the applicability of the RIs to other populations. However, the adult RIs for TSH and FT4 are consistent to that reported previously for a cohort from a different UK region on a Roche Cobas analyser.^10^ This suggests the potential for transferability of these derived ranges between laboratories using Roche assays. The limited number of reference participants above 70 years prevented us from exploring whether there was benefit from further age stratification of reference intervals in this age group.

In conclusion, we have found that thyroid tests are most commonly requested in primary care in people over the age of 60 years and specific RIs for TSH and FT4 for the over 60s lead to a reduction in abnormal thyroid test results in comparison to use of a single adult RI. This study adds to recent work demonstrating the potential benefit of age-related thyroid RIs for people of older age.^14,18,20,21^ Introduction of an over 60s RI would result in fewer people of older age being classified as subclinical hypothyroid or with marginally low FT4 that would consequently require regular clinical review and blood tests that may not be necessary or beneficial.

## Supplemental material

Supplemental Material - Age-related reference intervals for thyroid function tests are beneficial in adults over 60 years Supplemental Material for Age-related reference intervals for thyroid function tests are beneficial in adults over 60 years by George F. G. Allen, Sarah Blampied, Kashyap A. Patel, Timothy J. McDonald, Andrew T. Hattersley and Bijay Vaidya in Annals of Clinical Biochemistry.

## Data Availability

Reference cohort data can be accessed by application to the Peninsula Research Bank. Ethical approval currently restricts access to local researchers and their collaborators. See https://exetercrfnihr.org/about/exeter-10000-prb/

## References

[1] Brabant G Beck-Peccoz P Jarzab B , et al. Is there a need to redefine the upper normal limit of TSH? Eur J Endocrinol 2006; 154: 633–637. 16645008 10.1530/eje.1.02136

[2] Razvi S Jabbar A Addison C , et al. Variation in the reference range limits of thyroid function tests and association with the prevalence of levothyroxine treatment. Eur J Endocrinol 2023; 188: K5–K9. 10.1093/ejendo/lvad016 36751726

[3] Waise A Price HC . The upper limit of the reference range for thyroid-stimulating hormone should not be confused with a cut-off to define subclinical hypothyroidism. Ann Clin Biochem 2009; 46: 93–98. 19164339 10.1258/acb.2008.008113

[4] NICE. NICE Guidelines . NG145. Thyroid disease: assessment and management. https://www.nice.org.uk/guidance/ng145 (2019, accessed 29 April 2025).

[5] Pearce SHS Brabant G Duntas LH , et al. 2013 ETA guideline: management of subclinical hypothyroidism. Eur Thyroid J 2014; 2: 215–228. 10.1159/000356507 PMC3923601 24783053

[6] Hobbs FDR Bankhead C Mukhtar T , et al. Clinical workload in UK primary care: a retrospective analysis of 100 million consultations in England, 2007–14. Lancet 2016; 387: 2323–2330. 27059888 10.1016/S0140-6736(16)00620-6 PMC4899422

[7] Feakins B Oke J McFadden E , et al. Trends in kidney function testing in UK primary care since the introduction of the quality and outcomes framework: a retrospective cohort study using CPRD. BMJ Open 2019; 9: e028062. 10.1136/bmjopen-2018-028062 PMC6575820 31196901

[8] Javaid U Kennedy D Addison C , et al. Frequency, determinants and costs of thyroid function testing in a laboratory serving a large population. Eur J Endocrinol 2022; 186: 553–560. 35275844 10.1530/EJE-21-1172

[9] Ceriotti F Hinzmann R Panteghini M . Reference intervals: the way forward. Ann Clin Biochem 2009; 46: 8–17. 19103955 10.1258/acb.2008.008170

[10] Barth JH Luvai A Jassam N , et al. Comparison of method-related reference intervals for thyroid hormones: studies from a prospective reference population and a literature review. Ann Clin Biochem 2018; 55: 107–112. 28081637 10.1177/0004563217691549

[11] Horn PS Pesce AJ . Reference intervals: an update. Clin Chim Acta 2003; 334: 5–23. 12867273 10.1016/s0009-8981(03)00133-5

[12] Spencer CA Hollowell JG Kazarosyan M , et al. National Health and Nutrition Examination Survey III thyroid-stimulating hormone (TSH)-thyroperoxidase antibody relationships demonstrate that TSH upper reference limits may be skewed by occult thyroid dysfunction. J Clin Endocrinol Metab 2007; 92: 4236–4240. 17684054 10.1210/jc.2007-0287

[13] O’Leary PC Feddema PH Michelangeli VP , et al. Investigations of thyroid hormones and antibodies based on a community health survey: the Busselton thyroid study. Clin Endocrinol 2006; 64: 97–104. 10.1111/j.1365-2265.2005.02424.x 16402936

[14] Razvi SS Wild H Ingoe L , et al. Changes in thyroid function and autoimmunity in older individuals: longitudinal analysis of the Whickham cohort. J Clin Endocrinol Metab 2024; 110: e3078–e3084. 10.1210/clinem/dgae875 PMC12342363 39673773

[15] Surks MI Hollowell JG . Age-specific distribution of serum thyrotropin and antithyroid antibodies in the U.S. population: implications for the prevalence of subclinical hypothyroidism. J Clin Endocrinol Metab 2007; 92: 4575–4582. 17911171 10.1210/jc.2007-1499

[16] Stott DJ Rodondi N Kearney PM , et al. Thyroid hormone therapy for older adults with subclinical hypothyroidism. N Engl J Med 2017; 376: 2534–2544. 28402245 10.1056/NEJMoa1603825

[17] de Montmollin M Feller M Beglinger S , et al. L-thyroxine therapy for older adults with subclinical hypothyroidism and hypothyroid symptoms: secondary analysis of a randomized trial. Ann Intern Med 2020; 172: 709–716. 32365355 10.7326/M19-3193

[18] Yamada S Horiguchi K Akuzawa M , et al. The impact of age- and sex-specific reference ranges for serum thyrotropin and free thyroxine on the diagnosis of subclinical thyroid dysfunction: a multicenter study from Japan. Thyroid 2023; 33: 428–439. 36772798 10.1089/thy.2022.0567 PMC10620437

[19] Ross DS . Treating hypothyroidism is not always easy: when to treat subclinical hypothyroidism, TSH goals in the elderly, and alternatives to levothyroxine monotherapy. J Intern Med 2022; 291: 128–140. 34766382 10.1111/joim.13410

[20] Jansen HI Dirks NF Hillebrand JJ , et al. Age-specific reference intervals for thyroid-stimulating hormones and free thyroxine to optimize diagnosis of thyroid disease. Thyroid 2024; 34: 1346–1355. 39283820 10.1089/thy.2024.0346

[21] Li Q Tang Y Yu X , et al. Thyroid function reference intervals by age, sex, and race: a cross-sectional study. Ann Intern Med 2025; 178: 921–929. 40324200 10.7326/ANNALS-24-01559

[22] Taylor PN Lansdown A Witczak J , et al. Age-related variation in thyroid function – a narrative review highlighting important implications for research and clinical practice. Thyroid Res 2023; 16: 7. 37009883 10.1186/s13044-023-00149-5 PMC10069079

[23] Exeter clinical research facility. Exeter 10,000. https://exetercrfnihr.org/about/exeter-10000/ (accessed 21 May 2025).

[24] Rodgers LR Hill AV Dennis JM , et al. Choice of HbA1c threshold for identifying individuals at high risk of type 2 diabetes and implications for diabetes prevention programmes: a cohort study. BMC Med 2021; 19: 184. 34412655 10.1186/s12916-021-02054-w PMC8377980

[25] R Core Team . R: a language and environment for statistical computing. https://www.R-project.org (2024, accessed 12 May 2025).

[26] Horowitz GL Altaie S Boyd J . EP28-A3c: defining, establishing, and verifying reference intervals in the clinical laboratory; approved Guideline—Third edition. Clinical and Laboratory Standards Institute 2010; 28: 12.

[27] Demers LM Spencer CA . Laboratory support for the diagnosis and monitoring of thyroid disease. Washington DC: National Academy of Clinical Biochemistry, 2002. https://myadlm.org/science-and-research/practice-guidelines/thyroid-disease (accessed 12 May 2025).

[28] Davison AC Hinkley DV . Bootstrap methods and their applications. Cambridge: Cambridge University Press, 1997.

[29] Canty A Ripley B . Boot: bootstrap R (S-Plus) functions. R package version 1.3-31, 2024.

[30] Wickham H . ggplot2: elegant graphics for data analysis. New York: Springer-Verlag, 2016.

[31] Somwaru LL Rariy CM Arnold AM , et al. The natural history of subclinical hypothyroidism in the elderly: the cardiovascular health study. J Clin Endocrinol Metab 2012; 97: 1962–1969. 22438233 10.1210/jc.2011-3047 PMC3387427

[32] Hollowell JG Staehling NW Dana Flanders W , et al. Serum TSH, T4, and thyroid antibodies in the United States population (1988 to 1994): National Health and Nutrition Examination Survey (NHANES III). J Clin Endocrinol Metab 2002; 87: 489–499. 11836274 10.1210/jcem.87.2.8182

[33] Kahapola-Arachchige KM Hadlow N Wardrop R , et al. Age-specific TSH reference ranges have minimal impact on the diagnosis of thyroid dysfunction. Clin Endocrinol 2012; 77: 773–779. 10.1111/j.1365-2265.2012.04463.x 22703566

[34] Surks MI Ortiz E Daniels GH , et al. Subclinical thyroid disease: scientific review and guidelines for diagnosis and management. JAMA 2004; 291: 228–238. 14722150 10.1001/jama.291.2.228

[35] Parle JV Franklyn JA Cross KW , et al. Prevalence and follow-up of abnormal thyrotrophin (TSH) concentrations in the elderly in the United Kingdom. Clin Endocrinol 1991; 34: 77–84. 10.1111/j.1365-2265.1991.tb01739.x 2004476

[36] Vadiveloo T Donnan PT Cochrane L , et al. The Thyroid Epidemiology, Audit, and Research Study (TEARS): the natural history of endogenous subclinical hyperthyroidism. J Clin Endocrinol Metab 2011; 96: E1–E8. 20926532 10.1210/jc.2010-0854

[37] Thayakaran R Adderley NJ Sainsbury C , et al. Thyroid replacement therapy, thyroid stimulating hormone concentrations, and long term health outcomes in patients with hypothyroidism: longitudinal study. BMJ 2019; 366: I4892. 10.1136/bmj.l4892 PMC6719286 31481394

