# Supplementary material for: Age-related reference intervals for thyroid function tests are beneficial in adults over 60 years
Source: Ann Clin Biochem. 2026 Feb 9;63(5):394–402. doi: 10.1177/00045632261426852 (PMC13550734; doi:10.1177/00045632261426852)
Supplement: Supplementary file 1 [file sj-pdf-1-acb-10.1177_00045632261426852.pdf]

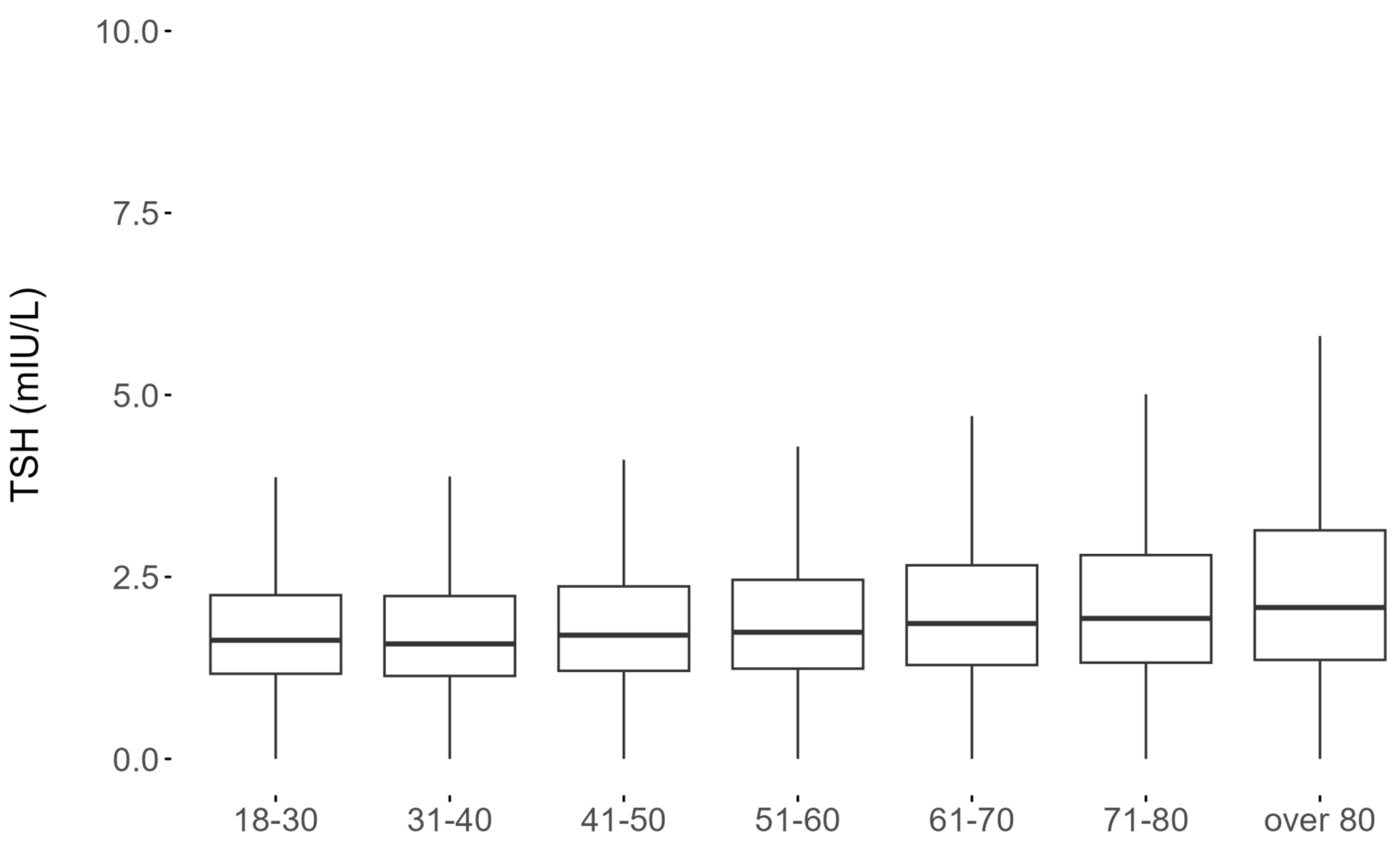

Supplementary figure 1: Effect of age on median serum thyroid stimulating hormone (TSH) concentration in the clinical cohort of 21,286 patients with no known thyroid disease. Age groups in years: 18-30 n = 1772; 31-40 n = 2262; 41-50 n = 2645; 51-60 n = 3860; 61-70 n = 3690; 71-80 n = 3958; over 80 n= 3099. *P* values are presented separately in supplementary table 1.

Supplementary table 1: Comparison of median thyroid stimulating hormone (TSH) values between age groups in the clinical cohort of 21,286 patients with no known thyroid disease. *P* values from Dunn's post hoc test with Bonferroni correction following Kruskal-Wallis test (overall *P* <0.0001)

	Comparison of median TSH *P* Values					
Age Group (y)	18-30	31-40	41-50	51-60	61-70	71-80
31-40	1.0000					
41-50	0.3404	0.0047				
51-60	0.0002	<0.0001	0.7420			
61-70	<0.0001	<0.0001	<0.0001	0.0001		
71-80	<0.0001	<0.0001	<0.0001	<0.0001	0.2354	
Over 80	<0.0001	<0.0001	<0.0001	<0.0001	<0.0001	0.0002

Supplementary table 2: Comparison of median TSH values between age and sex groups in the clinical and reference cohorts. *P* values from Dunn's post hoc test with Bonferroni correction following Kruskal-Wallis test.

Cohort	Age Group	Sex	n	Median TSH (IQR)	*P* value
Clinical	18-60	Female	7217	1.66 (1.18 – 2.35)	1.0000
		Male	3322	1.69 (1.22 – 2.37)	
	Over 60	Female	6520	1.91 (1.27 – 2.82)	<0.0001
		Male	4227	1.99 (1.40 - 2.85)	
Reference	18-60	Female	496	1.82 (1.27 – 2.39)	1.0000
		Male	552	1.86 (1.38 – 2.39)	
	Over 60	Female	163	2.04 (1.39 – 2.68)	1.0000
		Male	153	1.89 (1.48 – 2.48)	

Supplementary table 3: Comparison of median free thyroxine (FT4) and free triiodothyronine (FT3) values between age and sex groups in the reference cohort. *P* values from Dunn's post hoc test with Bonferroni correction following Kruskal-Wallis test. 2.5th and 97.5th centiles presented with bootstrap 95% confidence intervals.

	Age Group	Sex	n	Median (IQR)	*P* value	2.5th Centile (95% CI)	97.5th Centile (95% CI)
FT4 (pmol/L)	18-60	Female	496	15.2 (14.1 – 16.4)	<0.0001	12.2 (11.5, 12.4)	19.1 (18.8, 19.8)
		Male	552	15.7 (14.5 – 17.0)		12.3 (11.8, 12.7)	19.6 (19.0, 20.8)
	Over 60	Female	163	15.1 (14.0 – 16.6)	1.0000	11.6 (10.5, 12.5)	18.6 (17.8, 20.0)
		Male	153	15.2 (14.1 – 16.7)		11.2 (10.2, 12.3)	19.1 (18.3, 20.2)
FT3 (pmol/L)	18-60	Female	496	4.7 (4.4 – 5.0)	<0.0001	3.8 (3.7, 3.9)	6.1 (5.8, 6.3)
		Male	552	5.3 (4.9 – 5.6)		4.1 (4.0, 4.3)	6.5 (6.4, 6.6)
	Over 60	Female	163	4.6 (4.3 – 4.9)	0.0015	3.8 (3.5, 4.0)	5.7 (5.5, 5.9)
		Male	153	4.8 (4.6 – 5.2)		4.0 (3.8, 4.2)	5.9 (5.7, 6.6)

Supplementary table 4: Reference Intervals for serum thyroid stimulating hormone (TSH), free thyroxine (FT4) and free triiodothyronine (FT3) by age group with bootstrap 95% confidence intervals.

		TSH (mIU/L)		FT4 (pmol/L)		FT3 (pmol/L)	
Age Group	n	2.5th Centile (95% CI)	97.5th Centile (95% CI)	2.5th Centile (95% CI)	97.5th Centile (95% CI)	2.5th Centile (95% CI)	97.5th Centile (95% CI)
18-30	127	0.59 (0.46, 0.89)	4.92 (3.72, 5.31)	12.7 (12.2, 13.4)	21.2 (19.6, 21.6)	4.4 (4.0, 4.5)	7.2 (6.5, 7.6)
31-40	233	0.67 (0.41, 0.86)	3.81 (3.47, 4.70)	12.2 (10.9, 13.1)	19.2 (18.8, 20.3)	4.0 (3.7, 4.1)	6.5 (6.3, 6.6)
41-50	367	0.68 (0.62, 0.78)	3.91 (3.56, 4.18)	12.3 (11.9, 12.7)	19.2 (18.8, 19.8)	3.8 (3.7, 4.0)	6.2 (6.0, 6.4)
51-60	321	0.68 (0.58, 0.80)	4.75 (3.81, 5.23)	12.1 (11.4, 12.4)	18.9 (18.4, 19.4)	3.9 (3.8, 4.0)	5.9 (5.8, 6.0)

Supplementary table 5: Minimal overall effect of stratified age-specific reference intervals (RIs) for 18-60 year olds on result classification of thyroid stimulating hormone (TSH) from the clinical cohort (n = 10,539). LRL: Lower Reference Limit; URL: Upper Reference Limit. Age-specific RIs displayed in supplementary table 3.

		No. results per TSH category					
		Age Group (y)					
RI	TSH Category	18-30	31-40	41-50	51-60	Total	%
age-specific RI	TSH Below LRL	52	109	114	195	470	4.5
	TSH above URL	32	126	146	142	446	4.2
	TSH within RI	1688	2027	2385	3523	9623	91.3
all adult RI	TSH Below LRL	73	97	97	174	441	4.2
	TSH above URL	51	81	112	196	440	4.2
	TSH within RI	1648	2084	2436	3490	9658	91.6
